# Effects of extraction methods on protein properties obtained from paddy rice and germinated paddy rice

**DOI:** 10.7717/peerj.11365

**Published:** 2021-05-04

**Authors:** Wirot Likittrakulwong, Pisit Poolprasert, Khongsak Srikaeo

**Affiliations:** 1 Animal Science Program, Faculty of Food and Agricultural Technology, Pibulsongkram Rajabhat University, Muang, Phitsanulok, Thailand; 2 Biology Program, Faculty of Science and Technology, Pibulsongkram Rajabhat University, Muang, Phitsanulok, Thailand; 3 Food Science and Technology Program, Faculty of Food and Agricultural Technology, Pibulsongkram Rajabhat University, Muang, Phitsanulok, Thailand

**Keywords:** Rice protein, Alkaline extraction, SDS reagent, Enzymatic extraction, Protein property

## Abstract

Rice protein has attracted considerable attention recently due to its physiological effects. This study extracted the proteins from paddy rice (PR) and germinated paddy rice (GPR) using three methods i.e., alkaline, sodium dodecyl sulfate (SDS) reagent and enzymatic extractions. The extracted proteins or protein fractions were assessed for their properties using various techniques. Data were analyzed by 2′3 factorial design experiment. It was found that germination and extraction methods significantly affected the concentration of protein fractions when analyzed by Bradford assay. Average protein fraction concentration of the GPR was lower than that of PR. SDS-PAGE patterns of protein fractions obtained from PR and GPR using any extraction method displayed similar protein profiles. Three major protein bands at about 13 kDa (prolamin), 22–23 kDa (basic glutelin) and 37–39 kDa (acidic glutelin) with small amount of 57 kDa proglutelin were observed. For amino acid profile, germination increased the content of most amino acids, resulting in the higher content of amino acids in GPR, excepted for some amino acids. When processed with in vitro digestion, protein fractions from GPR exhibited a higher level of digestibility than those from PR as evidenced by the less intensity of the protein bands obtained from SDS-PAGE. Alkaline and SDS reagent extractions provided more digestible protein fractions than enzymatic extraction. Extraction methods also influenced phase transition of protein fractions as investigated by a DSC. Alkaline extraction resulted in protein fractions with higher phase transition temperature than the other methods. For antioxidant capacity, extraction methods as well as germination significantly affected antioxidant capacity of the protein fractions. Enzymatic extraction provided protein fractions with the best antioxidant capacity.

## Introduction

Rice is one of the most important cereal crops with high consumption rate in many parts of the world (Zargarchi & Saremnezhad, 2019). Brown rice or un-milled rice contains more nutritional components than milled or polished rice. However, they are rarely consumed as a staple food despite their elevated content of bioactive components. This is due to their dark appearance and hard texture when compared to polished rice (Ohtsubo et al., 2005). To overcome this constraint, germination has been introduced. The germination of rice seeds can be used to improve the taste, texture and further enhance nutritional values and health functions (Wu et al., 2013). During germination, hydrolytic enzymes are activated, and high molecular weight polymers are hydrolyzed, causing the increase of oligosaccharides, amino acids and other bio-functional substances (Sirisoontaralak et al., 2015). Gamma-aminobutyric acid (GABA) is one of the important bio-functional substances produced during germination of rice. Therefore, GABA has been the topic extensively investigated (Patil & Khan, 2011).

Both brown rice and paddy rice can be used for germination. However, the most used raw material is brown rice. Only few published works have studied paddy rice as raw material for germination (Jirapa et al., 2016; Maisont & Narkrugsa, 2010; Zargarchi & Saremnezhad, 2019). Though, germination of paddy rice would be more effective, than that of brown rice (Moongngarm & Saetung, 2010).

Germinated rice has gained significant attention recently as germination enhanced eating quality and promoted health functions. It has become popular among health-conscious consumers because of its beneficial bioactive compounds, especially gamma-aminobutyric acid (GABA) (Sirisoontaralak et al., 2015). GABA which has a significant role in neurotransmission, is one of the important bio-functional substances produced during germination of rice. Therefore, GABA has been the topic extensively investigated (Patil & Khan, 2011).

There is a lack of information with regards to the change of proteins in germinated rice. Despite the fact that protein is one of the two most abundant components of the rice grain after starch. Bran and endosperm are parts of the rice grains that contain proteins. Endosperm proteins are mostly storage proteins, located in protein bodies between the starch granules (Agboola, Ng & Mills, 2005). Rice proteins are considered valuable because they are colorless, rich in essential amino acids, possess a bland taste, and are hypoallergenic (Chrastil, 1992). Despite the interest of rice proteins as a functional ingredient, there has been limited studies to date. This is because rice contains less proteins and the water solubility of rice proteins is also low. These make them difficult to extract and incorporate into food products (Shih, 2003). However, the awareness of health benefits of plant proteins has been increasing. Hence, several extraction methods e.g., alkaline, enzymatic and physical treatments for the purification of rice proteins are being investigated and applied industrially (Fabian & Ju, 2011). Rice proteins are increasingly being used as functional ingredients in various health products (Amagliani et al., 2017a).

The purpose of this study was to characterize the protein fractions extracted from germinated paddy rice (GPR) and paddy rice (PR) using different extraction methods. The information obtained from this study could add up the knowledge and enhance the utilization of rice proteins.

## Materials and Methods

### Materials and preparations

PR (RD 31 variety) was obtained from Phitsanulok province (Thailand). The GPR samples were prepared following the method described earlier (Likittrakulwong et al., 2020). Briefly, 10 kg of paddy was soaked with water (1:2 w/v) at room temperature for 12 h, in which the water was changed every 4 h to avoid fermentation, and then drained. Soaked paddy was spread in a plastic basket with the maximum thickness of 2-cm and then covered with damp cloths maintaining the relative humidity of 90–95% by regular spraying of water. The germination took about 24 h. The GPR was dried at 50 °C in a tray-dryer, to approximately 10% of moisture content. Dried GPR was ground using a laboratory mill to pass a 100-mesh screen. Dried and ground PR without germination was used as the control sample for comparison. All chemicals and enzymes were analytical grade purchased from Sigma-Aldrich (Thailand) Co., Ltd.

### Protein extraction methods

Protein fractions were extracted using three different methods including alkaline, detergent sodium dodecyl sulfate (SDS) reagent and enzymatic extractions.

For alkaline extraction, the samples were extracted following the method described earlier (De Souza et al., 2016) with some modifications. NaOH solution (0.18% NaOH 1,500 mL) in a beaker was acclimated to 30 °C using a temperature controlled hotplate with capable of magnetic stirring. Then, 100 g of the ground samples was added to the solvent, kept under stirring for 30 min and then centrifuged at 3,380 ×g for 5 min. The supernatant (protein part) was separated for further analysis. The residue (starchy part) was washed with 250 mL of 0.18% NaOH and centrifuged at 3,380×g for 5 min. The washing extract was added to the extracted protein obtained previously. The collected extracts were used for further analysis.

For SDS reagent, the 10% SDS (w/v) solution was prepared by dissolving SDS in PBS (Phosphate Buffered Saline) (1X, pH 7.4). The ground rice samples were incubated with 0.5 mL of water and 0.5 mL of a 10% SDS solution, providing a detergent per sample ratio of 10:1 (w/w). The mixtures were incubated at 100 °C for 5 min to enhance SDS-protein solubilization (Mota et al., 2018). After which, samples were centrifuged, and the supernatant was separated and used for further analysis.

In terms of enzymatic extraction, starch degradation enzymatic method for rice protein extraction was applied following the method described earlier (Kumagai et al., 2006) with some modifications. The ground samples (100 g) were mixed with a heat-resistant *α*-amylase solution, 0.6 g porcine *α*-amylase (Sigma Cat#A3176) in 1 L of water. The mixture was stirred at 80 °C for 30 min and heated to 90 °C for 10 min. After lowering the temperature below 50 °C, the mixture was centrifuged at 3,000 g for 10 min to collect the precipitate. The precipitate was treated with the heat-resistant *α*-amylase solution again. The precipitate which represented the extracted protein fractions was washed with phosphate buffer saline and freeze dried (Lyovapor™ L-300, Switzerland). The freeze-dried extract was used for further analysis.

### Quantification and identification of proteins

#### Bradford assay

The concentration of protein fractions was measured using the Bradford method (Bio-Rad Protein Assay Cat# 500-0006, Bio-Rad Laboratories Ltd., Thailand). Briefly, the Coomassie assay reagent was mixed with protein samples at a 1:1 volume ratio and then incubated for 10 min at room temperature. The measurements were carried out according to the manufacturer’s instruction.

#### SDS-PAGE

The extracted protein fractions from different extraction methods were characterized by sodium dodecyl sulfate polyacrylamide gel electrophoresis (SDS-PAGE) analysis. All protein fractions were suspended in sample buffer (0.125 M Tris–HCl, pH 6.8, 4% w/v SDS, 5% v/v 2-mercaptoethanol, 4 M urea, heated to 100 °C for 3 min, and subjected to SDS-PAGE analysis on 15% (w/v) polyacrylamide gels as described elsewhere (Yang et al., 2007).

#### Amino acid analysis

Amino acid analysis of the extracted protein fractions was performed using a Biochrom 30+ amino acid analyzer (Biochrom Ltd., UK) following the manufacturer’s instruction.

#### In vitro digestion

The samples were defatted by chloroform–methanol Soxhlet extraction. The in vitro digestion of the extracted protein fractions with pepsin and pancreatin was performed according to the method described elsewhere (Yang et al., 2012) with slight modification. Using the defatted samples, protein solution, 5% w/v in distilled water, was adjusted to pH 2.0 with 1 M HCl. Porcine pepsin (1%, w/w, protein basis) (Sigma Cat#77160) was added and the system was incubated at 37 °C. During the digestion process, the digesta was sampled at the intervals of 45, 90 and 135 min. Deproteinization of the digesta was performed by using 30% trichloroacetic acid (TCA). The free amino group, which was reacted with 2,4,6-trinitrobenzenesulfonic acid at 37 °C for 2 h, was measured spectrophotometrically at 420 nm. After 2 h of pepsin digestion, the digesta was adjusted to pH 8.5 with NaHCO_3_, and porcine pancreatin (3%, w/w, protein basis) (Sigma Cat#P1750) was added. The mixture was incubated at 37 °C for 8 h. The digesta was sampled at the times of 45, 90, 135 and 180 min, treated with 30% TCA and centrifuged at 12000g for 5 min at room temperature. After centrifugation, the acid-soluble fraction was subject to SDS-PAGE analysis as described earlier.

### Thermal properties

The extracted protein fractions from PR and GPR were freeze dried and ground into powder by the ultra-centrifugal mill and sieved through a 100-mesh screen. DSC experiment was conducted using a Mettler Toledo DSC 1 equipped with a refrigerated cooler following the conditions previously reported (Amagliani et al., 2016). The samples were weighted (30 mg) directly into 120 µL medium pressure crucibles. The samples were tempered at 5 °C for 5 min and heated to 100 °C at a heating rate of 5 °C min^−1^. A pan containing 30 mg calcined aluminium oxide was used as a reference. Peak temperature (*Tp*) and enthalpy (Δ*H*) of protein denaturation was determined using the STAR^e^ software (Mettler Toledo, USA).

### Antioxidant capacity

For antioxidant capacity, protein fractions solution was prepared by dissolution in deionized water at the appropriate dilution.

Total phenolic compounds were analyzed using a modified Folin-Ciocalteau colorimetric method (Singleton, Orthofer & Lamuela-Raventos, 1999). Briefly, an aliquot of the 40-fold water diluted protein fractions extract (0.2 mL) was added to a 15 mL tube and 0.2 mL of 1:10 Folin-Ciocalteau reagent:water solution was added. The tube was kept at room temperature for 1 min and then two mL of 7.5% (w/v) Na_2_CO_3_ was added, incubated at room temperature for 2 h. The absorbance of the incubated samples was measured at 765 nm. The results expressed as gallic acid equivalent (GAE)/g protein, using calibration curves of gallic acid as the standard.

The ability to donate hydrogen to the free radical DPPH and the ferric reduction antioxidant power (FRAP) were determined as described elsewhere (Benzie & Strain, 1996; Brand-Williams, Cuvelier & Berset, 1995). The results expressed as Trolox equivalent (TE)/g protein, using calibration curves of Trolox as the standard.

### Statistical analysis

Experiment was conducted using the 2 × 3 factorial design in CRD. Paddy (PR and GPR) and extraction methods (alkaline, SDS reagent and enzymatic extractions) were considered as the factors. Hence, there were 6 experimental treatments (T) assigned as follows; T1 = PR and SDS reagent, T2 = PR and alkaline extraction, T3 = PR and enzymatic extraction, T4 = GPR and SDS reagent, T5 = GPR and alkaline extraction, T6 = GPR and enzymatic extraction. Data are presented as the mean values (triplicate) and analyzed using full factorial analysis. The criterion for significance was *P* < 0.05.

## Results

### Quantification and identification of proteins

#### Protein concentration

Bradford assay was used to quantify the concentration of protein fractions isolated from PR and GPR and they are shown in Table 1. Generally, both factors (paddy and extraction methods) significantly affected the protein quantities (*P* < 0.05). Average protein fraction concentration of the GPR was found to be lower than that of PR. Enzymatic extraction provided the highest protein fraction concentration and it was statistically different (*P* < 0.05) from SDS reagent and alkaline extractions.

**Table 1:** Protein concentration as determined by Bradford assay and statistical analysis results.

Treatments	Protein concentration (g/100g dry sample)
T1 = PR and SDS reagent	45.97ab
T2 = PR and alkaline extraction	43.14cd
T3 = PR and enzymatic extraction	47.364a
T4 = GPR and SDS reagent	40.79e
T5 = GPR and alkaline extraction	42.13de
T6 = GPR and enzymatic extraction	44.60bc
Pooled SEM	0.10
**Main effect means for paddy**	
PR	45.49a
GPR	42.51b
**Main effect means for extraction**	
SDS	43.38b
Alkaline	42.64b
Enzymatic	45.98a
***P* value**	
Paddy (PR, GPR)	<0.01
Extraction (SDS, Alkaline, Enzymatic)	0.003
Paddy × Extraction	0.035

**Notes.**

Means from triplicate analysis. Means with the different letters are significantly different (*P* < 0.05).

#### SDS-PAGE

**Figure 1:**
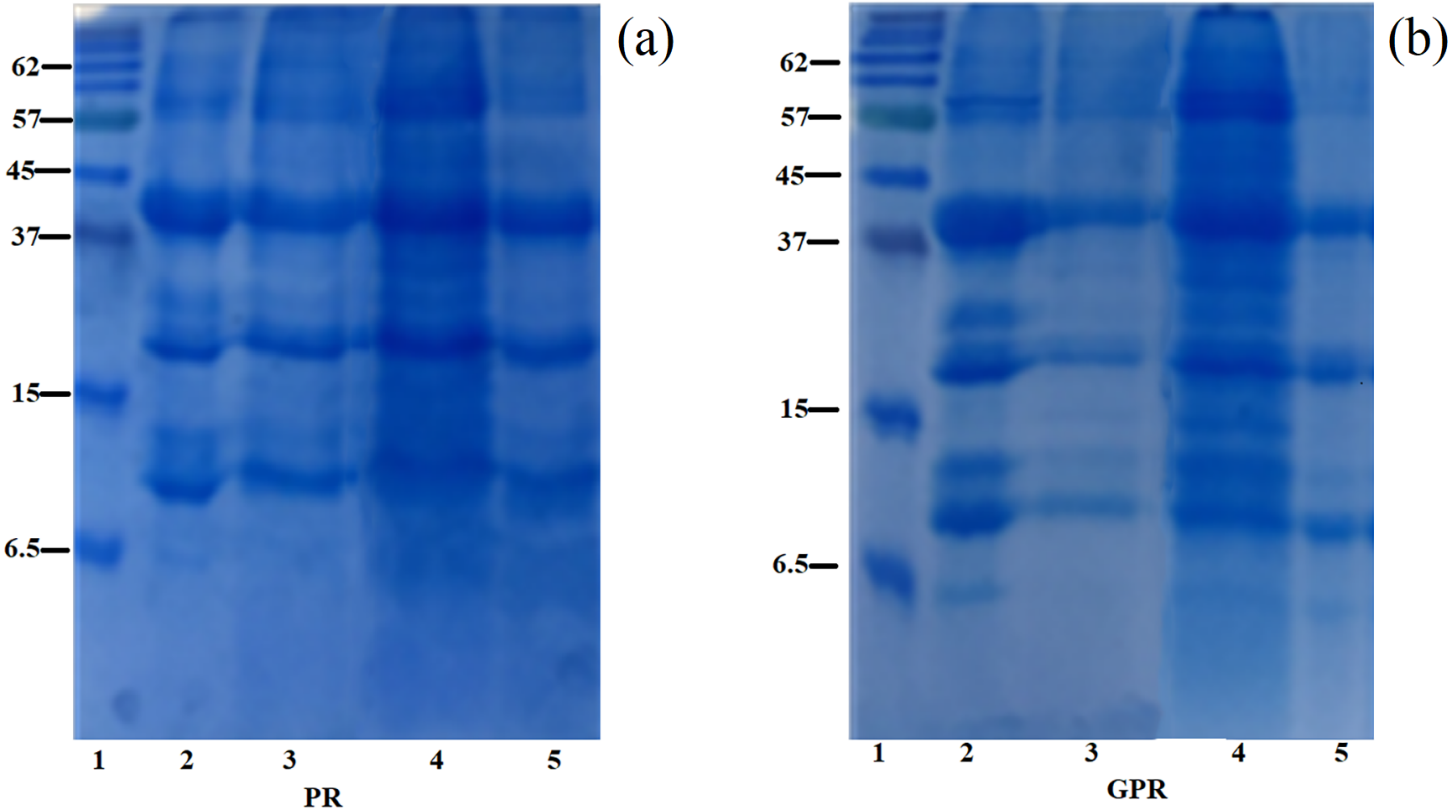
SDS-PAGE patterns of proteins extracted from PR (A) and GPR (B) in comparison with rice flour (RF). Lane1: molecular weight marker with sizes in kDa indicated on the left (Protein Ladder, Bio-Rad), Lane 2: RF, Lane 3: alkaline extraction, Lane 4: SDS reagent extraction, Lane 5: enzymatic extraction.

SDS–PAGE showing the information of the polypeptide composition of rice protein fractions obtained from PR and GPR is shown in Fig. 1. In this study, PR and GPR exhibited similar protein profiles regardless of the extraction methods as clearly seen in Fig. 1A for PR and Fig. 1B for GPR. Rice protein composition and its molecular masses varied depending on the solubility fractions which related to rice variety and the extraction procedures. For RF (Lane 2), our study found that it displayed three main bands at about 13 kDa (prolamin), 22–23 kDa (basic glutelin) and 37–39 kDa (acidic glutelin) with small amount of 57 kDa proglutelin. For PR and GPR, three major bands were observed in both PR and GPR with the same range of molecular weight as determined in RF sample. However, germination seemed to cause the reduction of polypeptides as evidenced by the SDS-PAGE patterns especially in GPR extracted using alkaline and enzymatic extractions, Fig. 1B.

#### Amino acid analysis

Amino acids composition of PR and GPR is reported in Table 2. Generally, germination increased the content of most amino acids, resulting in the higher content of amino acids in GPR, excepted for aspartic acid, glutamic acid, methionine and histidine. No significant differences were observed for threonine and alanine.

**Table 2:** Amino acid composition (mg/g) of paddy rice (PR) and germinated paddy rice (GPR).

Amino acid	PR	GPR
Asp	16.32 ± 0.40a	15.93 ± 0.42b
Glu	34.71 ± 0.91a	30.07 ± 0.81b
Lys	7.84 ± 0.06b	11.07 ± 3.44a
Ser	9.76 ± 0.23b	10.46 ± 0.27a
Arg	17.68 ± 0.51b	18.89 ± 0.59a
Gly	14.99 ± 0.37b	15.06 ± 0.40a
Thr	3.88 ± 0.09a	3.93 ± 0.11a
Ala	8.88 ± 0.21a	8.89 ± 0.22a
Pro	15.94 ± 0.37b	18.04 ± 0.49a
Val	13.83 ± 0.35b	14.08 ± 0.19a
Met	2.84 ± 0.08a	2.37 ± 0.06b
Ile	30.50 ± 0.76b	32.32 ± 0.86a
Leu	16.64 ± 1.22b	17.72 ± 1.36a
Tyr	16.23 ± 0.57b	20.86 ± 0.54a
His	11.67 ± 0.24a	8.34 ± 0.47b
Phe	14.00 ± 0.25b	16.40 ± 0.34a

**Notes.**

Means ± SEM from triplicate analysis. Means with the different letters are significantly different (*P* < 0.05) as evaluated by *t*-test.

Germination contributed to the enhance of rice protein nutritional quality. In this study, essential amino acids such as lysine, leucine, isoleucine, threonine, phenylalanine and valine showed a significant increase in GPR when compared to PR (Table 2).

In addition, the germination brought metabolic changes to GPR. Proteins are breakdown into amino acids especially glutamic acid which converted into GABA by the action of glutamate decarboxylase. GABA content was significantly increased with germination.

#### In vitro digestion

**Figure 2:**
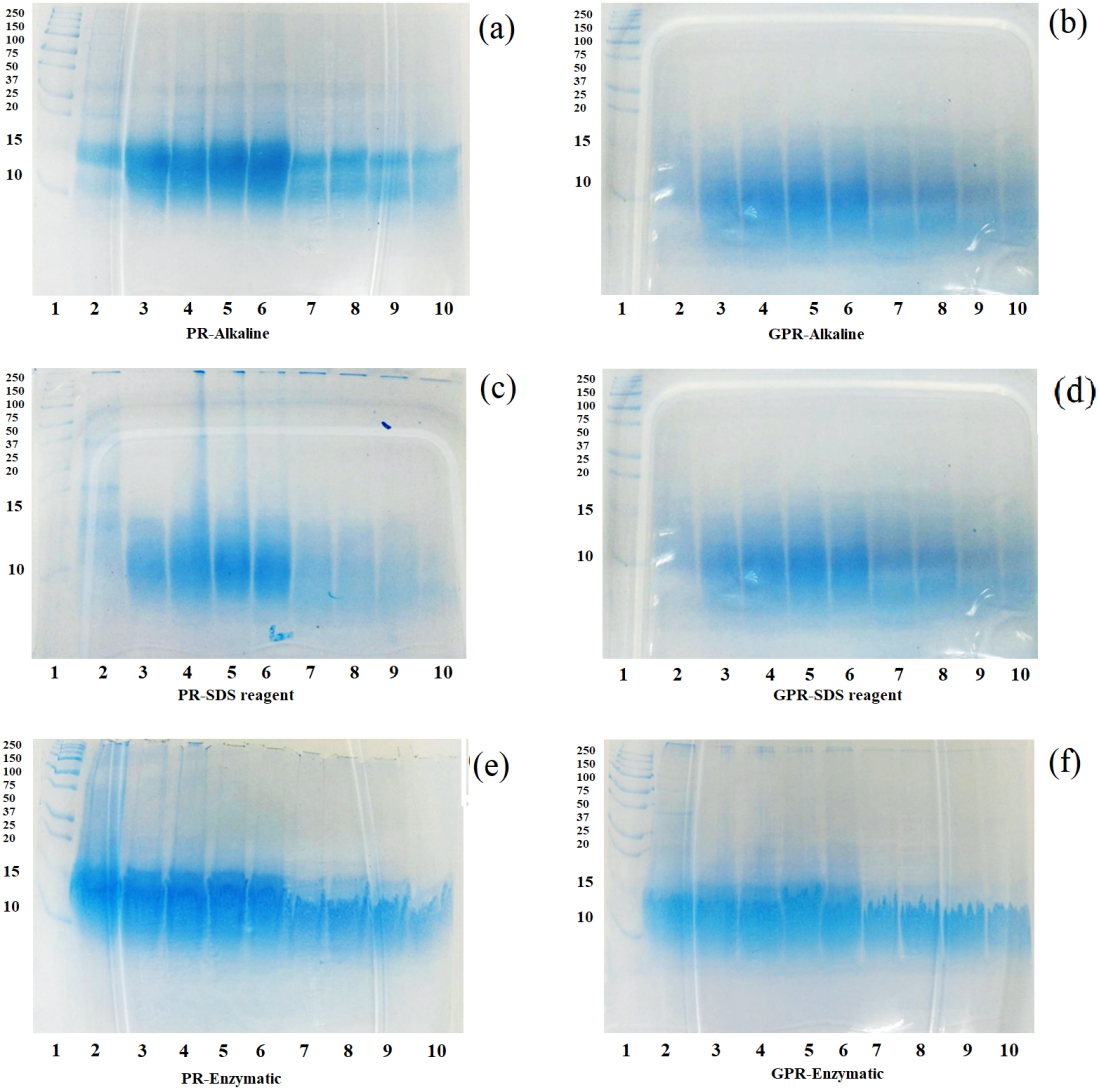
SDS-PAGE patterns of proteins extracted from PR (A, C, E) and GPR (B, D, F) after in-vitro digestion. Lane1: molecular weight marker with sizes in kDa indicated on the left (Protein Ladder, Bio-Rad), Lane 2: undigested sample, Lanes 3–6: pepsin digestion at 30, 60, 90 and 120 min respectively, Lanes 7–10: pancreatin digestion at 30, 60, 90 and 120 min respectively.

After the in vitro digestion by pepsin and pancreatin, the in vitro digestibility of protein fractions from PR and GPR were determined by SDS-PAGE and the patterns are shown in Figs. 2A–2F. In general, GPR (Figs. 2B, 2D and 2F) exhibited a higher level of digestibility than that of PR (Figs. 2A, 2C and 2E) as evidenced by the less intensity of the protein bands. Germination resulted in the changes of bioactive compounds and increased nutritional values. Rice protein fractions from both PR and GPR were able to be digested by pepsin from 30 min onwards and they were more digested after pancreatin digestion. Protein bands at around 10–15 kDa remained visible throughout the digestion period, though they decreased the intensity during pancreatin digestion. Rice protein fractions from both PR and GPR with high molecular weights were completely digested after pancreatin digestion as evidenced by the absence protein bands. Alkaline and SDS reagent extractions provided more digestible proteins as they exhibited less intensity of protein bands when compared to enzymatic extraction.

### Thermal properties

Thermal properties of the extracted rice protein fractions as investigated by the DSC showing phase transition temperatures and enthalpies are shown in Table 3. DSC was employed for characterization of the thermal properties of the extracted protein fractions. The peak temperature (*Tp*) and enthalpy (Δ*H*) of protein denaturation were observed from the single endothermic peak found in all samples, ranging from 78−96 °C with small enthalpy energies (Table 3). The denaturation (phase transition) peak temperature can be used as an indicator of the thermal stability of proteins. While the enthalpy of the denaturation is related to the proportion of undenatured proteins or the extent of ordered protein structure to the denatured proteins.

**Table 3:** Peak temperature (*Tp*) and enthalpy (Δ*H*) of protein denaturation as examined by DSC.

Treatments	*Tp* (° C)	Δ*H* (J g^−1^)
T1 = PR and SDS reagent	78.41	0.53
T2 = PR and alkaline extraction	91.99	0.17
T3 = PR and enzymatic extraction	80.54	0.16
T4 = GPR and SDS reagent	78.80	0.55
T5 = GPR and alkaline extraction	95.72	0.12
T6 = GPR and enzymatic extraction	79.41	0.14
Pooled SEM	2.142	0.057
**Main effect means for paddy**		
PR	83.64	0.29
GPR	84.64	0.27
**Main effect means for extraction**		
SDS	78.60b	0.54a
Alkaline	93.85a	0.14b
Enzymatic	79.97b	0.15b
***P* value**		
Paddy (PR, GPR)	0.390	0.643
Extraction (SDS, Alkaline, Enzymatic)	<0.01	<0.01
Paddy × Extraction	0.248	0.786

**Notes.**

Means from triplicate analysis. Means with the different letters are significantly different (*P* < 0.05).

Germination did not affect phase transition statistically (*P* ≥ 0.05), refers Table 3. However, extraction methods influenced phase transition statistically (*P* < 0.05). Alkaline extraction resulted in protein fractions with higher phase transition temperature. This indicated that alkaline extracted protein fractions were resistant to heat. For enthalpy, all samples showed small enthalpy changes with less than 1 J g^−1^, while SDS reagent extracted proteins exhibited higher enthalpy energy than the others, indicating that its protein fractions were less denatured. Protein compositions in the extracted protein fractions play a major role in control the thermal properties. Different extraction methods provided different protein composition and consequently affected their thermal properties.

### Antioxidant capacity

Antioxidant capacity of rice proteins as investigated by total phenolic compounds, DPPH and FRAP is shown in Table 4. Generally, types of paddy (PR and GPR) as well as extraction methods significantly affected antioxidant capacity (*P* < 0.05). Their interactions are also statistically significant (*P* < 0.05). PR and GPR exhibited significant difference (*P* < 0.05) in antioxidant capacity, providing different values of total phenolic compounds, DPPH and FRAP. Total phenolic compounds and FRAP values of GPR are higher than those of PR. Although, DPPH values showed differently. In terms of extraction methods, it was found that extraction methods significantly affected (*P* < 0.05) antioxidant capacity of the proteins. Enzymatic extraction provided the best antioxidant capacity as evidenced by high values of total phenolic compounds, DPPH and FRAP. While alkaline extraction exhibited the least antioxidant capacity.

**Table 4:** Antioxidant capacity as examined by total phenolic compounds, DPPH and FRAP assays.

Treatments	Total phenolic compounds (µgGAE/g)	DPPH (µgTE/g)	FRAP (µgTE/g)
T1 = PR and SDS reagent	0.74c	0.32b	2.70cd
T2 = PR and alkaline extraction	0.04d	0.02d	3.17bc
T3 = PR and enzymatic extraction	1.78b	0.44a	2.30de
T4 = GPR and SDS reagent	0.76c	0.19c	3.53ab
T5 = GPR and alkaline extraction	0.07d	0.07d	2.08e
T6 = GPR and enzymatic extraction	2.90 a	0.13c	3.96a
Pooled SEM	0.305	0.043	0.205
**Main effect means for paddy**			
PR	0.85b	0.26a	2.72b
GPR	1.24a	0.13b	3.19a
**Main effect means for extraction**			
SDS	0.75b	0.25a	3.12a
Alkaline	0.05c	0.05b	2.62b
Enzymatic	2.34a	0.29a	3.13a
**P value**			
Paddy (PR, GPR)	0.001	<0.01	0.007
Extraction (SDS, Alkaline, Enzymatic)	0.003	<0.01	0.021
Paddy × Extraction	0.035	<0.01	<0.01

**Notes.**

Means from triplicate analysis. Means with the different letters are significantly different (*P* < 0.05).

## Discussion

### Quantification and identification of proteins

#### Protein concentration

The component of protein in rice is relatively low, but the total amount of rice protein potentially available is significant because the production of rice worldwide is huge. In this study, the extraction yield obtained from all extraction methods was approximately 7% by weight, similar to previously reported (7–9% by weight). Rice proteins are good quality proteins and their products have been in demand. However, because of difficulties in the processing, rice protein products, particularly high-protein content ones, have not been readily available (Shih, 2003).

Alkaline, enzymatic and physical treatments have been evaluated for their extraction potential, and some have been applied industrially (Amagliani et al., 2017b). Proteins from cereals including rice have been commonly extracted using alkaline solutions (De Souza et al., 2016). In this study, enzymatic extraction provided better results than alkaline and SDS reagent extractions. It should be emphasized that enzymatic protein extraction processes have some drawbacks e.g., complex process control, long processing time, high operational costs, high energy consumption, and irreversible carbohydrate-protein matrix disruption. However, it is considered a mild and green extraction method (Sari, Bruins & Sanders, 2013). In addition, it produces superior products which are more suitable for human consumption (Amagliani et al., 2017b). Different enzymes have been applied for rice protein extractions and their extraction efficiencies depended on many factors such as enzyme types and the concentrations used for extraction (Hanmoungjai, Pyle & Niranjan, 2002).

#### SDS-PAGE

Generally, albumin, globulin, glutelin and prolamin have been reported and they accounted for 4.5%, 13.1%, 79.7% and 2.6% respectively in rice flour sample (Ju, Hettiarachchy & Rath, 2001). Molecular masses of albumin, globulin, glutelin and prolamin fractions were reported to be distributed in the range of 30–45, 20–66, 10–66 and 10–53 kDa, respectively (Adebiyi et al., 2009). Though, others reported different values (Hamada, 1997). Rice albumin was found to resolve into a wide range of subunits, with molecular weight ranging from about 13 to 110 kDa, and globulin to be between 16 and 130 kDa (Amagliani et al., 2017a). Various other studies on rice proteins also reported different relative molecular mass values for the various protein fractions. These discrepancies are assumed to be caused by the heterogeneous nature of polypeptides in rice (Ghanghas et al., 2020).

Three main bands i.e., prolamin, basic glutelin and acidic glutelin with small amount of proglutelin were identified for RF in this study which were in agreement with previous published reports (Amagliani et al., 2017a; De Souza et al., 2016; Wang et al., 2016). In addition, extraction methods showed small impact on PR and GPR protein profiles. It has been reported earlier that alkaline and enzymatic extractions did not affect the rice protein profile (Wang et al., 2016). Previous report was conducted in germinated brown rice and they found that the accumulation of all the major polypeptides, including prolamins, glutelin acidic and basic subunits was lower in germinated brown rice as compared to brown rice (Pal et al., 2016). The reduction in all major polypeptides could associate with the increase of proteases activity during germination (Kim et al., 2012).

#### Amino acid profiles

Amino acid profiles of PR and GPR were similar to the previous report (Moongngarm & Saetung, 2010). They found that germination significantly increased the content of almost all the amino acids, except histidine, methionine and threonine, glutamic acid, aspartic acid and serine. Special concerns should be made on the increase of some essential amino acids in germinated rice in which these amino acids are limited in ungerminated rice. Germination contributed to the enhance of rice protein nutritional quality especially glutamic acid which converted into GABA by the action of glutamate decarboxylase during germination. Our previous work on PR and GPR showed that GABA increased almost ten times after germination (Likittrakulwong et al., 2020). This trend agreed with several previous studies (Moongngarm & Saetung, 2010; Pal et al., 2016; Sibian, Saxena & Riar, 2017).

#### In vitro digestion

Extractions were reported to affect the digestibility of rice proteins. Alkaline extraction increased rice protein digestibility (Kumagai et al., 2006) while enzymatic extraction provided proteins with high resistant to pepsin-pancreatin digestion (Wang et al., 2016). Germination induced the changes of bioactive compounds and enhanced nutritional values. Therefore, GPR exhibited a higher level of protein digestibility than that of PR.

### Thermal properties

Denaturation temperatures as evidenced by DSC peaks were reported previously and varied depending on the protein composition (Amagliani et al., 2016). Peak transition temperature of rice albumin, globulin and glutelin were found to be 73.3, 78.9 and 82.2 °C, respectively, but no endothermic peak was observed for rice prolamin (Ju, Hettiarachchy & Rath, 2001). Proteins extracted from rice endosperms were reported to exhibit phase transition at 85.6 ° C (Zhao et al., 2012). Phase transition of protein fractions extracted from PR and GPR in this study are in agreement with previously reported. It has been reported that high amount of glutelin in rice proteins could increase transition temperature (Ju, Hettiarachchy & Rath, 2001).

### Antioxidant capacity

This study confirmed the previous findings in which extraction was a vital processing step to modify the antioxidant capacities of rice proteins. In vitro digestion steps also resulted in less efficacious antioxidant of rice proteins (Wang et al., 2016). In addition, germination which enhanced nutritional qualities of GPR, also enhanced its antioxidant capacity. It should be noted that the determination of the total phenolic content in this study employed the Folin-Ciocalteu’s protocol, the presence of all reducing substances in the samples such as polypeptides, amino acids, non-amino acid polyphenols etc. could affect the results (Ciulu, Cádiz-Gurrea & Segura-Carretero, 2018).

## Conclusions

Rice proteins extracted from PR and GPR using alkaline, SDS reagent and enzymatic extraction methods exhibited different properties. Germination enhanced nutritional qualities of rice seeds and this also contributed to protein qualities extracted from GPR. Extraction methods played a major role in controlling the properties of the extracted proteins. The present study provided the information on various properties of rice proteins extracted from both PR and GPR. These included Bradford assay protein concentration, SDS-PAGE profiles of the extracted proteins, amino acid contents, in vitro digestion, thermal properties, and antioxidant capacities. The findings are useful for characterization of the proteins extracted from paddy rice of which the research is still limited when compared to milled rice and rice bran. Paddy in both ungerminated and germinated forms could be used as a source for rice protein extraction.

##  Supplemental Information

10.7717/peerj.11365/supp-1 Supplemental Information 1 Raw data.

## Additional Information and Declarations

### Data Availability

The following information was supplied regarding data availability: The raw data before statistical analysis are available in the Supplemental File.
